# Incidents of Animal Life

**Published:** 1887-01-22

**Authors:** F. O. Morris

**Affiliations:** Rector of Nunburnholme, Yorkshire; author of "British Birds," etc.


### INCIDENTS OF ANIMAL LIFE.

COLLATED BY THE REV. F. O. MORRIS, B.A.,

Rector of Nunburnholme, Yorkshire; author of "British Birds," etc.

"He prayeth best, who loveth best
All things both great and small;
For the dear God who loveth us,
He made and loveth all."—COLERIDGE.

### STORIES ABOUT DOGS.

Introductory.

THE following anecdote of a dog is by far the most extraordinary that has ever come before me; and I have had more of such than most people, or, perhaps, than any one. I published "A Thousand-and-One Stories from Nature" in the *Fireside Magazine*, and very many hundreds more in my "Anecdotes in Natural History," "Records of Animal Sagacity," the "Christ Evangelist," "Land and Water," etc. The one I have now to give, was first printed in the "Anecdotes in Natural History," many years ago, and has since been out of print for several years, so that I feel sure it will be new to probably every one of your readers: in fact, I had quite forgotten that there had been such a story, myself, or was only reminded of it by a letter from a stranger, Mr. Duncan Guilding, writing first from Builth, in Breconshire, December 9th, 1886, and since from Llandovery, in Carmarthenshire. In his first note, inquiring if I was the author of the book, he wrote "I have tried to get it several times, through the ordinary channels, but have been met with the reply 'out of print.'" In his second, December 31st, he said, "I am given to understand that you quote in this book an anecdote relating to a terrier dog having returned without a guide from the interior of America to its home in Gloucestershire. This dog belonged to a relation of mine, and my father was one of the party who went out at the time the dog was taken, and it was to his parish that the latter returned. I have often heard my father—who is now seventy-one years of age—relate the tale. We wonder how you got hold of it. If your memory serves you, would you mind telling me how you picked up this anecdote?" This I could not do, though, on referring to the book itself, I soon found it, for I saw that I had prefaced it with the following remark: "I have omitted unfortunately to note the name of the writer of the next account, but that it is worthy of credit I am sure, or I should not have narrated it." Thereon comes the story itself, as follows:—

A Strange Story.

"On the 19th of May, 1834, a party who had been living at Quedgeley, within two miles of Gloucester, sailed from Bristol to New York, intending to settle in one of the Western States of America. They took with them a wire-haired terrier, which whelped during the passage. The distance from Quedgeley to Bristol is twenty-seven miles. From New York, they proceeded in a steam-boat up the Hudson to Albany, 190 miles; thence to Schenectady, fifteen miles, by railroad; to Syracuse, 140 miles, by tow-boat. In the hurry of disembarking at Syracuse, the dog was missed, and all trace of her lost. Some time after arriving at their destination, one of the party wrote to his father, and amongst other things, mentioned the loss of the dog, which animal, at the moment the letter arrived at Quedgeley, was lying down in front of the kitchen fire of the house which she had been originally taken from, having been absent from her original home ten months. It is conjectured (for nothing is known) that she found her way back to New York, and thence to Bristol, but how, or in what ship, is a matter of doubt; that she did make this extraordinary tour is beyond the slightest question. She ended her days in a drain at Quedgeley, being smothered whilst rat-hunting there in 1841. She was the property of Mr. Richard Guilding, formerly of Quedgeley, who went to St. Louis, in the State of Mississippi, and returned from thence to Hanley Castle, Worcestershire, at which place he is now residing."

